# “Phthalates”: Kelley et al. Respond

**DOI:** 10.1289/ehp.1205763R

**Published:** 2012-11-01

**Authors:** Katherine E. Kelley, Erica L. Chaplin, Allen A. Mitchell, Sonia Hernández-Díaz, Russ Hauser

**Affiliations:** 1 Slone Epidemiology Center at Boston University, Boston, Massachusetts, E-mail: kekelley@bu.edu; 2 Department of Epidemiology, Harvard School of Public Health, Boston, Massachusetts; 3 Department of Environmental Health, Occupational and Environmental Medicine and Epidemiology Program, Harvard School of Public Health, Boston, Massachusetts

We appreciate Carter’s review of our article (Kelley et al. 2012) and his further clarification of the chemistry across this class of compounds. In the “Results” of our article, we specifically noted the use of *ortho*-phthalates (DBP and DEP), which were often found to be used in combination with the phthalate polymers he discussed in his letter [cellulose acetate phthalate (CAP), hypromellose phthalate (HMP), and polyvinyl acetate phthalate (PVAP)]. We believe that we appropriately distinguished the difference between the *ortho*-phthalates and the polymers, specifically noting that the polymers have “no known toxicity.”
